# A new haemocyanin in cuttlefish (*Sepia officinalis*) eggs: sequence analysis and relevance during ontogeny

**DOI:** 10.1186/2041-9139-5-6

**Published:** 2014-02-05

**Authors:** Anne Thonig, Michael Oellermann, Bernhard Lieb, Felix Christopher Mark

**Affiliations:** 1 Integrative Ecophysiology, Alfred Wegener Institute for Polar and Marine Research, 27570 Bremerhaven, Germany; 2 Institute of Zoology, Johannes Gutenberg University of Mainz, 55099 Mainz, Germany

**Keywords:** Respiratory protein, Cephalopods, Mollusks, Embryogenesis, Haematopoiesis, Development

## Abstract

**Background:**

Haemocyanin is the respiratory protein of most of the Mollusca. In cephalopods and gastropods at least two distinct isoforms are differentially expressed. However, their physiological purpose is unknown. For the common cuttlefish *Sepia officinalis,* three isoforms are known so far, whereas for only two of them the complete mRNA sequences are available. In this study, we sequenced the complete mRNA of the third haemocyanin isoform and measured the relative expression of all three isoforms during embryogenesis to reveal a potential ontogenetic relevance.

**Results:**

The cDNA of isoform 3 clearly correlates to the known *Sepia officinalis* haemocyanin subunits consisting of eight functional units and an internal duplicated functional unit d. Our molecular phylogenetic analyses reveal the third isoform representing a potentially ancestral haemocyanin isoform, and the analyses of the expression of haemocyanin type 3 reveal that haemocyanin type 3 only can be observed within eggs and during early development. Isoforms 1 and 2 are absent at these stages. After hatching, isoform 3 is downregulated, and isoform 1 and 2 are upregulated.

**Conclusions:**

Our study clearly shows an embryonic relevance of the third isoform, which will be further discussed in the light of the changes in the physiological function of haemocyanin during ontogeny. Taken together with the fact that it could also be the isoform closest related to the common ancestor of cuttlefish haemocyanin, the phylogeny of cuttlefish haemocyanin may be recapitulated during its ontogeny.

## Background

Haemocyanin is the respiratory protein of arthropods and molluscs. This copper-containing pigment occurs freely dissolved in the haemolymph of these animals and is coloured blue when oxygenated. The haemocyanins of both mentioned phyla have a copper type-3 oxygen-binding site, but otherwise show structural differences and evolved separately [1-3]. The basal quaternary structure of molluscan haemocyanins is a cylindrical decamer composed of ~350–400 kDa large subunits (SU) [4]. In Polyplacophora and Cephalopoda, only decamers can be observed floating in the haemolymph [5,6]. In Bivalvia and Gastropoda, they are also able to form didecamers and multidecamers [7,8]. The single subunits usually consist of 7–8 tandemly arranged functional units (FU) of ~50 kDa each. Every FU exhibits one oxygen-binding site [1]. These paralogous FUs evolved by subsequent gene/exon duplications and fusions [9,10]. The subunits of cephalopod haemocyanins are characterised by the lack of the most C-terminal FUh [6,10]. The 350 kDa haemocyanin subunits of Tetrabranchiata and Octobrachia consist of 7 FUs (a-g), contrary to the 8 FUs (~400 kDA) in Decabrachia [1,11]. For the common cuttlefish (*Sepia officinalis*) and Spirula (*Spirula spirula*), it has been shown that a duplication of FUd led to the 8 FUs (a-b-c-d-d’-e-f-g; [12,13]).

Besides the duplications of single FUs, duplications of the complete haemocyanin gene occurred as well. This happened several times independently in different groups and resulted in different isoforms of the protein [4]. Gastropods and bivalves can feature one or two isoforms [1,8,14-20], while in cephalopods probably only the tetrabranchiate *Nautilus pompilius* is restricted to a single haemocyanin [6]. The recent coleoid cephalopods were all found to express at least two different isoforms of the respiratory protein (e.g., *Sepia officinalis*, DeGeest *et al*., unpubl.; *Allotheuthis media*, Oellermann *et al*., unpubl.; *E. dofleini*[9]). So far, the complete mRNA sequences of haemocyanin isoform 1 (SoH1 [GenBank:DQ388569]) and isoform 2 (SoH2 [GenBank:DQ388570]) have been identified in *Sepia officinalis*. Next to these two known isoforms there is great evidence for a third one in the cuttlefish (SoH3; [21,22]), whereof one fragment of ~1,000 bp has already been sequenced [GenBank:JN392726]. The existence of these isoforms raises questions about their evolution referring to origin and structural changes as well as to their physiological relevance.

Due to its moderate evolutionary rate, haemocyanin has been used to estimate the divergence time of different groups among Mollusca, e.g., the most recent common ancestor of cephalopods, Dibranchiata and Decabrachia was calculated [13]. Additionally, the duplication of the *S. officinalis* isoforms could be dated to ~90 million years ago (Mya), whereas the isoforms of *E. dofleini* split much more recently. In both isoforms of *S. officinalis*—as well as in *S. spirula*—the duplicated FUd is present [11,13]. This points to a duplication event of FUd earlier than the split of the isoforms. Yet with no complete sequence of the third isoform, no further implications concerning the origin and evolutionary development of that isoform can be drawn.

However, all isoforms are expressed, and SoH1 and SoH2 even display different theoretical isoelectric points (*in silico*: SoH1: pI = 5.79, SoH2: pI = 5.85), which in turn could affect the oxygen affinity [21,22]. The existence of different isoforms might therefore serve a physiological purpose. Indeed, it has already been shown that some vertebrates and invertebrates can change the composition or concentration of their respiratory protein in response to changing environmental parameters such as temperature and oxygen partial pressure *P*O_2_ (*Gadus morhua*[23], *Daphnia magna* and *D. pulex*[24,25]; *Triops longicaudatus*[26], *Artemia franciscana*[27]). Moreover, cephalopods display the highest metabolic rates among invertebrates and therefore have to rely on an efficient oxygen supply, which is also indicated by the great Bohr effect of their haemocyanin [28-30]. Nevertheless, *S. officinalis* displays no difference in relative expression of the haemocyanin isoforms in response to changing temperature, hypercapnia or hypoxia [22, Thonig *et al.*, unpublished].

Besides adaptation to changing environmental conditions, different isoforms of respiratory proteins are also known to be expressed during the course of ontogeny (Crustaceans [31], *Cancer magister* and *C. productus*[32-35], *Artemia salina*[36], *Schistocerca americana*[37], *Chironomus plumosus* and *Chironomus thummi*[38,39], chicken [40] and mammals [41]). For example, humans start with the expression of embryonic haemoglobin, which is replaced by foetal, and afterwards by adult haemoglobin (e.g., [42]). Likewise, the maternal haemocyanin in the oocytes of the crustacean *Cancer magister* is subsequently replaced by embryonic, juvenile and adult haemocyanin [43].

Thus, the evolution of embryonic versions of the respective respiratory proteins (haemoglobin and haemocyanin) occurred independently in invertebrates and vertebrates. This might indicate common challenges referring to oxygen supply during early development. Therefore, an ontogenetic relevance of the different haemocyanin isoforms could be the case for *Sepia officinalis*. This is assumable especially as cuttlefish embryos have to face hypoxic and hypercapnic conditions towards the end of embryogenesis since their egg capsule functions as a diffusion barrier [44-46]. Actually, the existence of an embryonic haemocyanin in cuttlefish had already been proposed on the basis of electrophoretic distinct protein fractions of haemocyanin in embryos, juveniles and adults of *Sepia officinalis*[47,48]. Furthermore, Beuerlein *et al.*[49] identified haemocyanin mRNA [GenBank:AF401231] not only in the branchial glands of cuttlefish embryos, which would reflect the situation in adults, but also in haemocytes, the branchial hearts, the midgut gland and the renal appendages. This indicates both a switch of function in the haemocyanin metabolism of these tissues accompanied with hatching and a change of the site of synthesis of haemocyanin during ontogeny [50]. This would be well in line with a shift of haemocyanin expression. Finally, Strobel *et al.*[22] detected the highest relative expression of the recently found potential isoform 3 in newly hatched cuttlefish, while it decreased significantly with time after hatching. This could point out an embryonic function of haemocyanin isoform 3.

We therefore identified the complete sequence of haemocyanin isoform 3, and revealed its origin and evolutionary history. On the other hand, we tried to elucidate the role of this isoform 3 during embryogenesis by analysing the relative expression of the haemocyanin isoforms in different embryonic stages of the cuttlefish *Sepia officinalis*; in this way, we combined an evolutionary and physiological approach to characterise the new differentially expressed haemocyanin isoform 3 of *S. officinalis*.

## Methods

### Animal collection and sampling

Clusters of cuttlefish eggs were collected in May 2011 in Chioggia (Adria, Italy) and transferred to and kept at the Alfred Wegener Institute in Bremerhaven, Germany. For further investigations, the egg capsules were removed, and the length and width of the embryos as well as their developmental stages according to Lemaire [51] were determined. We distinguished five different stages: E1 (Lemaire 21–24), E2 (Lemaire 25–26), E3 (Lemaire 26–27), E4 (Lemaire 28–29) and H (Lemaire 30). Hereafter, either the complete embryos, the gills or the outer yolk sac were immediately frozen in liquid nitrogen for subsequent RNA analysis. Additionally, adult specimens collected in the English Channel (Caen) in 2010 and raised at the Alfred Wegener Institute at 15°C were used to compare the expression of haemocyanin mRNA (Thonig *et al.*, unpublished).

### cDNA synthesis

Due to the low weight of the individual embryonic and tissue samples, RNA was mainly extracted from pools of different individuals of the same developmental stages using the RNeasy Mini Kit (Qiagen GmbH, Hilden, Germany). The pools ranged from one to eight individuals corresponding to 9 to 102 mg input material (Table 1). The samples were homogenised for 15 s at 6,500 rpm in a Precellys beadmill (Bertin Technologies, Peqlab Biotechnologies GmbH, Erlangen, Germany) with doubled amount of recommended lysis buffer RLT (Qiagen GmbH, Hilden, Germany) at room temperature. Further extraction procedures followed the RNeasy Mini Kit protocol. A DNase treatment of each extract was carried out according to the Ambion Turbo DNA-free™ Kit (Ambion, Applied Biosystems, Darmstadt, Germany). Afterwards, cDNA synthesis was performed via RT-PCR with 0.4 μg of RNA either according to the High Capacity cDNA Reverse Transcription Kit (Applied Biosystems, Darmstadt, Germany) for Real-Time PCR or according to the Invitrogen Superscript III cDNA synthesis kit (Life Technologies) for subsequent sequencing of the haemocyanin isoform 3 cDNA.

**Table 1:** **Tissue and embryo pools of *S. officinalis *** **used for cDNA synthesis and subsequent real-time qPCR**

**Name**	**Stage (Lemaire)**	**Tissue**	**No. of individuals**	**Input material [mg]**	**Comment**
E1_Y	21-23	Yolk	2	102	No statistics
E1_E1	21-23	Embryo	8	49	
E1_E2	21-23	Embryo	2	13	
E2_E1	24-26	Embryo	3	45	
E2_E2	24-26	Embryo	2	25	
E2_E3	24-26	Embryo	2	28	
E3_E1	26-27	Embryo	2	43	
E3_E2	26-27	Embryo	2	45	
E3_E3	26-27	Embryo	1	29	
E3_E4	26-27	Embryo	1	34	
E3_E5	26-27	Embryo	2	48	
E3_E6	26-27	Embryo	1	31	
E4_E1	28-29	Embryo	2	19	
E4_E2	28-29	Embryo	1	47	
E4_E3	28-29	Embryo	1	32	
H_G	30	Gill	4	12	No statistics

### qPCR and statistical analysis

The relative expression of the different haemocyanin isoforms was analysed using an ABI 7500 Real-Time PCR System with the respective 7500 SDS Software, version 1.3 (Applied Biosystems, Darmstadt, Germany). The 20 μl PCR reaction was performed in MicroAmp® Optical 96 well plates (Applied Biosystems, Darmstadt, Germany) using the SYBR® Green PCR Master Mix (Applied Biosystems, Germany). Each qPCR replicate contained 2 ng of cDNA and isoform-specific primers with a final concentration of 300 nM. The primers were designed and optimised by Strobel *et al.*[22] on the basis of the respective sequences available in GenBank:

Isoform 1 [GenBank:DQ388569]:

SofHc1_fwd CTTTTCGAGTTTACCAGCTCTTGTT,

SofHc1_rev CCTGCAACGTCAATATATGAGTGAT.

Isoform 2 [GenBank:DQ388570]:

SofHc2_fwd TCTCCCGTTTTTGGTAACTGAAC,

SofHc2_rev TGTCAGCAACATCAATATAACCATGA.

Isoform 3 [GenBank:JN392726]:

SofHc3_fwd TGCTCCGTCAACCAATGTCC,

SofHc3_rev TCCAGGGCATCTCGTTTTCAC.

The PCR programme was composed of a 10 min activation step at 95°C and 40 amplification cycles (15 s at 95°C for denaturing, 1 min at 60°C for annealing and elongation) with a single fluorescence measurement during the annealing step. A melting curve analysis was performed to ensure the amplification of one single product. Efficiencies for the different primer pairs were calculated according to the equation E = 10^(−1/slope)^, using the decadic logarithm of cDNA input plotted against the cycle thresholds (C_T_) for five template dilution steps (efficiency SoH1: 1.843, SoH2: 1, 1.859, SoH3: 1.824 ).

For further analysis, the C_T_-values were transformed into relative quantities [52] using MS Excel according to the following equation: quantity (x) = efficiency ^(C^_T_^(Minimum)−C^_T_^(x))^. Furthermore, the relative quantities were normalised to adjust differences in template input using the normalisation coefficients calculated by geNorm [53]. For this reason, the potential housekeeping genes cytochrome c oxidase (COX) and nucleolin were analysed via Real-Time PCR as well. The respective primer pairs were designed on the basis of 454 pyrosequencing results of cDNA of the branchial gland of adult *Sepia officinalis* (Lieb & Mark, unpublished) using Primer Express Software for Real-Time PCR, version 3.0 (Applied Biosystems, Darmstadt, Germany) (nucleolin: Nuc_fw CAAGCCGCAAAGAAAGACAAG, Nuc_rv GCTTTAGATTCCTTTTTGGAAGCA, COX: COX_fw CAATAGGAGCCGTTTTTGCG, COX_rv AGGGTAATCAGAGTATCGTCGTGG) and yielded efficiencies of 1.719 for COX and 1.809 for nucleolin. The relative quantities of the respective C_T_ values of the potential housekeeping genes were then used to calculate the normalisation coefficients for the expression of the haemocyanin isoforms with geNorm.

Finally, Nalimov’s test [54] on a significance level of 95% was performed with the normalised relative quantities of the haemocyanin isoforms to remove outliers. The data were illustrated as the mean with standard error using GraphPad Prism 5.0b (GraphPad Software, Inc., La Jolla, CA, USA) and the expression trend using Adobe Illustrator (Adobe Systems Software Ireland Ltd., Ireland). Statistical analysis to determine significant differences in expression of the three haemocyanin isoforms during embryonic development was performed using the non-parametric Mann–Whitney U-Test via SPSS Statistics 19 (IBM, Ehningen, Germany).

### Sequencing

The SoH3 cDNA sequence was obtained via primer walking using cDNA derived from yolk and embryonic samples of the earliest developmental stage as template to ensure a large amount of SoH3 mRNA. Touchdown PCRs were performed with one degenerated primer coding for the conserved copper-binding sites [10] and one SoH3-specific primer designed with MacVector 12.0 (MacVector, Inc., Cambridge, UK) based on the already known fragment of SoH3. The touchdown PCR protocols consisted of a 4 min activation step at 94°C, 12 touchdown cycles with the annealing temperature decreasing by 1°C per cycle and finally 35 amplification cycles. The annealing temperature and elongation time of the PCR protocols were chosen with respect to the melting temperature of the primer pair and the length of the expected PCR product. The amplicons were separated in a 1.3% agarose gel with GelRed and extracted using the QIAQuick Gel Extraction Kit (Qiagen GmbH, Hilden, Germany). Afterwards, the extracts were either used for an additional amplification and purification step to increase product concentration or sequenced directly by Eurofins MWG GmbH (Martinsried, Germany).

Additionally, PCR products were obtained using two degenerated primers [10]. Those ones and PCR products giving double peaks after the sequencing reactions were cloned into the pGemT-easy vector (Promega, Madison, WI, USA) or TOPO TA vector (Life Technologies Invitrogen, Darmstadt, Germany). A PCR with the standard vector primer M13 uni and M13 rev was followed by an RFLP analysis using several restriction enzymes (MunI, HinfI, AluI, SpnI, MboI, RsaI) to determine different inserts. These inserts were then purified using the QIAQuick Gel Extraction Kit and sequenced by Eurofins MWG GmbH (Martinsried, Germany) or GATC Biotech AG (Konstanz, Germany).

The 5′-end and 3′- end of the SoH3 mRNA were amplified with the Ambion FirstChoice ®RLM-RACE Kit and the Invitrogen GeneRacer Kit (Life Technologies Invitrogen, Darmstadt, Germany) according to the manual and sequenced by Eurofins MWG GmbH (Martinsried, Germany) or GATC Biotech AG (Konstanz, Germany).

Additionally, genomic DNA was isolated from cuttlefish branchial glands using Qiagen DNeasy columns according to the manufacturer’s protocol. After shearing of 1 μg DNA (Covaris S2), an Illumina paired-end library was constructed according to TruSeq DNA Sample Preparation v2 protocol. First, the DNA was end repaired, 3′-ends were adenylated and adapters were ligated. Subsequently the DNA was amplified (10 cycles) and double size selected using the SPRIselect reagent kit (Beckman Coulter). The resulting DNA fragments ranged between 300 and 600 bp with a mean size of ca. 280 bp. Sequencing was done on 1.375 lanes of an Illumina HiSeq 2000 paired-end flowcell resulting in approximately 400,000,000 reads. DNA processing and sequencing were done by Genterprise (Mainz).

### Sequence analysis

The single sequences were trimmed, corrected on the basis of the chromatogram and assembled to a complete SoH3 sequence using Geneious 6.1.2 (Biomatters). Overlaps of at least ~150 bp were used to assure a correct assembly. The Illumina reads were trimmed and mapped on the cDNA sequence allowing maximum 10% mismatches per read. From this mapping the position of introns and the FUs could be deduced according to the GT/AG rule. The 5′- and 3′-UTRs were annotated according to the derived open reading frame (ORF). The respective protein sequence was obtained via translation using the standard genetic code. Furthermore, a signal peptide was detected via SignalP v4.1 and potential N-glycosylation sites using NetNGlyc1.0. Finally, the molecular weight as well as the theoretical isoelectric point of haemocyanin subunit 3 was determined via ExPASy Bioinformatics Resource Portal [55].

For phylogenetic analyses the haemocyanin sequences of *Sepia officinalis* (SoH1 [GenBank:DQ388569], SoH2 [GenBank:DQ388570]), *Enteroctopus dofleini* (A-type [GenBank:AY751301], G-type [GenBank:AF338426]), *Nautilus pompilius* [GenBank:AJ619741] and *Haliotis tuberculata* (HtH1 [GenBank:AJ252741], HtH2 [GenBank:AJ297475]) as outgroup were added from GenBank. For those sequences the FUs were annotated according to the genomic data of *S. officinalis*[6,10]. The multiple sequence alignments of the complete haemocyanin subunits and the FUd/d’ were obtained using MUSCLE implemented in Geneious 6.1.2. The alignments were curated using Gblocks 0.91b ([56]; parameters used for haemocyanin/FUd alignment: minimum number of sequences for a conserved position: 5, minimum number of sequences for a flanking position: 6/7, maximum number of contiguous nonconserved positions: 8, minimum length of a block: 10; allowed gap positions: none, use similarity matrices: yes) to eliminate poorly aligned positions. Subsequently, we determined the appropriate substitution model via ProtTest 3.2 [57] according to the corrected Akaike information criterion (AICc): WAG + I + G + F (I = 2.199, G = 0.199 for complete Hc sequences), WAG + G + F (G = 0.799 for FUd/d’). Based on these substitution models, Bayesian trees were calculated via MrBayes 3.2.1 [58] running an MCMC analysis with 2,000,000 generations and a sampling frequency of 200 generations. According to the respective trace files, the likelihoods had reached stationarity before 20% of the MCMC chain, which was therefore used as the burnin fraction. Additionally, maximum likelihood trees were calculated using the PhyML plugin of Geneious 6.1.2 [59] performing 1,000 replicates to calculate bootstrap values. Since the PhyML plugin did not allow to account for the amino acid frequencies (+F), the following substitution models were chosen: LG + I + G (I = 1.316, G = 0.144; for complete Hc sequences), WAG + I + G (I = 0.193, G = 1.696 for FUd/d’).

A relaxed molecular clock was calculated with BEAST v1.5.4 using a Bayesian MCMC analysis [60]. The following settings were chosen: WAG + I + G as substitution model, an uncorrelated log-normal distribution as clock mode and birth-death process as tree prior. Two analyses were run for 10,000,000 generations and were logged every 1,000 generation. Stationarity and appropriate mixing of each was checked using Tracer v1.5 [60]. The runs were combined using LogCombiner and the resulting file was processed with TreeAnnotator using a burnin of 20%. An additional run only under the priors was performed to confirm that the chosen priors were sufficiently non informative. The split of Gastropoda-Cephalopoda in the late Cambrian 550 ± 50 Mya was used as calibration point [6,7,17].

### Ethics statement

All sampling of cuttlefish was conducted according to the ethics and guidelines of the German law and do not require a formal permit. The experiments have been communicated according to § 8 animal welfare act (18.05.2006; 8081. I p. 1207) to the veterinary inspection office ‘Senatorin für Arbeit, Frauen, Gesundheit, Jugend und Soziales, Abt. Veterinärwesen, Lebensmittelsicherheit und Pflanzenschutz’, Bahnhofsplatz 29, 28195 Bremen, Germany, evaluated and approved of on July 12, 2011.

## Results

### Haemocyanin mRNA expression

**Figure 1:**
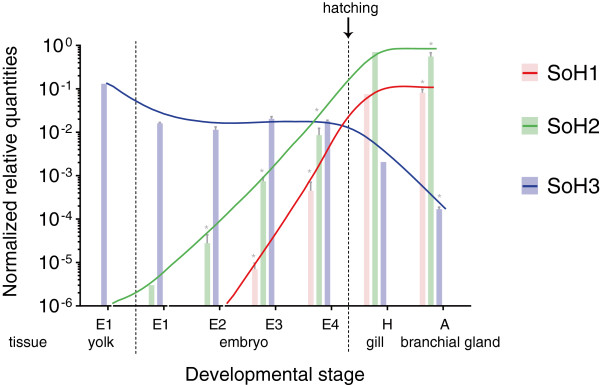
**Haemocyanin expression during ontogeny.** The mRNA expression of the different haemocyanin isoforms (SoH1, SoH2, SoH3) of *Sepia officinalis* in different tissues (yolk, embryo, gill, branchial gland) and developmental stages ranging from embryos (E1-E4) to hatchlings (H) and adults (A). The histogram shows the mRNA expression as normalised relative quantities with standard error. Thereby, asterisks indicate when the expression of the respective isoform differs significantly compared to the previous developmental stage on a significance level of 0.05 according to the Mann–Whitney-U test. Due to the low number of samples for E1 yolk and J gill, those stages are not taken into account for statistics. The lines illustrate the shift of the haemocyanin isoforms.

To investigate a potential ontogenetic relevance of the existence of different haemocyanin isoforms in cuttlefish, we measured their expression in different developmental stages of embryogenesis via Real-Time PCR. Thereby, we detected distinct differences during ontogeny referring to both the relative expression (Figure 1) and the ratio of the isoforms (Table 2). As shown in Figure 1, the mRNA of isoform 3 is highly expressed in embryos and also in the yolk sac of the earliest developmental stage. While SoH3 remains almost constantly at a high level of expression during embryonic development, significant amounts of SoH2 and SoH1 appear only during late organogenesis in stage E3 and E4, respectively. Though SoH2 could be detected in very low amounts already at stage E1 (even in the yolk sac) and E2, it only increased significantly during the following stages E3 and E4. Finally, SoH1 was first present at stage E3 with significantly increased expression shortly before hatching (E5). Thereby SoH3 represents about 99.8%–100% of the haemocyanin expressed in early organogenesis (E1 + E2) measured in the complete embryo (Table 2). Before hatching, SoH3 accounts for 66.6% of haemocyanin, while SoH2 represents 31.7% and SoH1 1.7%. In gills of hatchlings (H), ratios of the haemocyanin isoforms were measured as follows: SoH2 90.1%, SoH1 9.7% and SoH3 0.3%. This reflects the situation found in the branchial gland of adult cuttlefish (incubation at 15°C): SoH2 87.2%, SoH1 12.8% and SoH3 0.03%.

**Table 2:** Percentage of the different isoforms adding to haemocyanin mRNA

**Sample**	**SoH1**	**SoH2**	**SoH3**
E1_Y	0	0.001	99.999
E1_E	0	0.016	99.984
E2_E	0	0.249	99.751
E3_E	0.031	3.499	96.469
E4_E	1.657	31.73	66.613
H_G	9.674	90.062	0.264
A_BG	12.799	87.174	0.027

The percentage is given for the respective tissues (Y: yolk; E: embryo; G: gill; BG: branchial gland) of different ontogenetic stages ranging from embryos (E1-E4) to hatchlings (H) and adults (A).

### SoH3 sequence

The SoH3 mRNA sequence (GenBank:KF306341) consists in total of 10,277 bp. It is composed of a 39 bp 5′-UTR, a 10,017 bp ORF containing the eight FUs and a 221 bp 3′-UTR. The typical AATAAA polyadenylation signal can be found 13 bp upstream of the poly-(A) tail. Partly different versions of the haemocyanin sequence were obtained from sequencing and could not definitely be assigned referring to either the chromatogram or genomic data, or via cloning. Therefore, 20 ambiguous sites of 10,277 bp in total (representing 0.2%) remain in the sequence. They are mainly spread in the FUa (5), FUb (8) and FUc (7).

**Figure 2:**
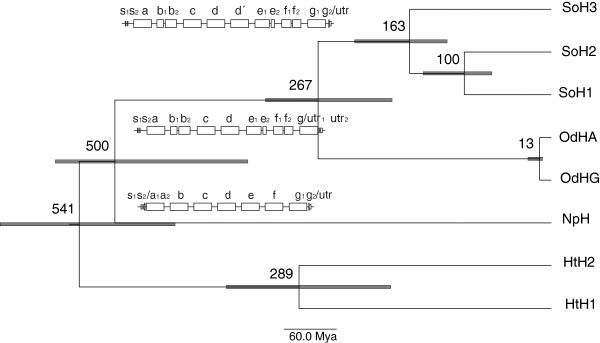
**Molecular clock and exon-intron structure of cephalopod haemocyanin.** Relaxed molecular clock with a mean evolutionary rate of 7.636 × 10^-4^ calculated with BEAST v1.5.4 based on a maximum likelihood tree for the amino acid sequences of the complete haemocyanin molecules of *Sepia officinalis* (SoH1-SoH3), *Enteroctopus dofleini* (OdHA, OdHG), *Nautilus pompilius* (NpH) and *Haliotis tuberculata* (HtH1, HtH2). The split of gastropoda and cephalopoda about 550 ± 50 Mya was used as calibration point. Included are the exon-intron structures of the haemocyanin of *N. pompilius*, *E. dofleini* and *S. officinalis.* Grey bars indicate the 95% HPD (highest posterior density), i.e. the Bayesian confidence interval of the estimated age.

**Figure 3:**
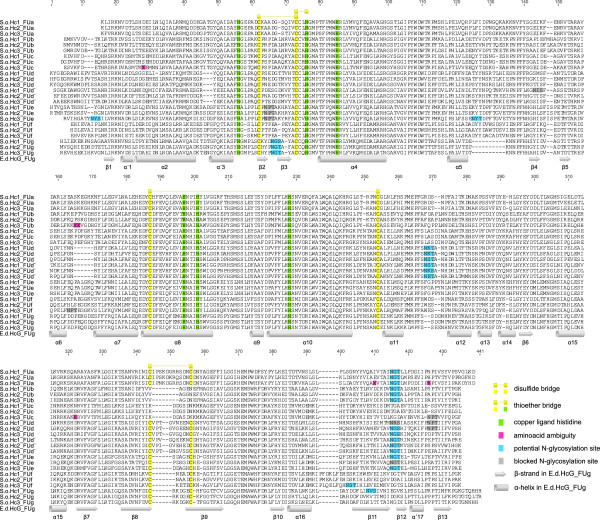
**Alignment of the three cuttlefish haemocyanins (SoH1-SoH3).** Highlighted are the characteristic six histidine residues forming the oxygen binding sites (green), one thioether bridge, three disulphide bridges and the potential glycosylation sites predicted with NetNGlyc1.0. The secondary structure of the FUg of *E. dofleini* (Cuff *et al*., 1998) is displayed on the bottom.

The deduced primary structure consists of 3,338 amino acids (aa), including a signal peptide of 26 aa and the actual polypeptide of 3,312 aa. This consists of 8 FUs ranging from 409 to 418 aa, reflecting the FUs found in the other known isoforms. Thus, the duplication of FUd/d’ is also present in SoH3. The translated sequence still contains 0.2% ambiguities (6 aa of 3,338 aa in total: FUa: 2, FUb: 2, FUc: 2), which all resemble isofunctional amino acid exchanges. The linker introns found between the FUs are all in phase 1. The internal introns in the signal peptide (phase 0), FUb (phase 2), FUe (phase 0) and FUf (phase 0) reflect the situation found in *E. dofleini* referring to location and phase [10], whereas the one found in FUg (phase 1) represents the internal intron also present in *N. pompilius*[6] (Figure 2). The haemocyanin protein SoH3 has a calculated molecular mass of 377,793 Da with a theoretical isoelectric point (pI) of 5.94. Each FU shows the characteristics for molluscan haemocyanin also found in *Enteroctopus* and *Nautilus*[6] (Figure 3). These are six histidine residues for copper binding (histidine residues in total: 173) as well as one thioether bridge (Cys73-His76) and three potential disulphide bridges (Cys62-Cys73, Cys187-Cys254, Cys344-Cys356), whereas the last one is not present in FUb and FUc. Ten potential glycosylation sites can be detected, nine NXT motifs and one NXS motif (Figure 3), whereas in FUc, FUd and FUe the central proline should block the glycosylation site [61]. The observed glycosylation pattern of SoH3 reflects the one already described for cuttlefish SoH1 [62] and cephalopods in general [6]: no glycosylation of FUc, one conserved glycosylation site (aa 415) in FUa, FUb, FUd, FUd’ and FUe, one in FUg (aa 66) and another one present in FUd and FUd’ (aa 268), which is probably not glycosylated in FUd’ [62]. Differences concerning the glycosylation appear in FUe and FUf, in which SoH3 exposes more potential sites for glycosylation (FUe: aa 14 and aa 125, FUf: aa 163 and aa 402). The amino acid composition between the isoforms does not differ considerably.

### Phylogeny of cephalopod haemocyanin

**Figure 4:**
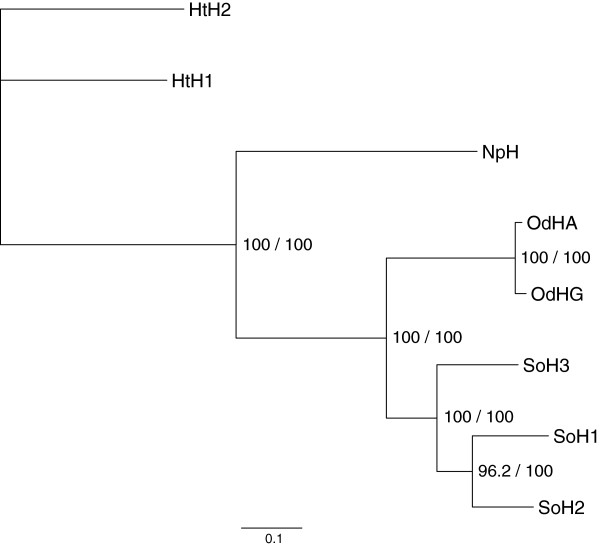
**Maximum likelihood tree of the amino acid sequences of haemocyanin.** The tree includes the haemocyanin sequences of *Sepia officinalis* isoform 1–3 (SoH1, SoH2, SoH3), *Enteroctopus dofleini* A-type (OdHA) and G-type (OdHG), *Nautilus pompilius* (NpH) and *Haliotis tuberculata* isoform 1 and 2 (HtH1, HtH2) calculated with PhyML using the LG + I + G model to illustrate the evolution of cephalopod haemocyanin. The tree topology of the Bayesian tree calculated with MrBayes using the WAG + I + G + F model is similar. Therefore, support for the tree nodes is given by the bootstrap values of 1,000 replicates and the consensus support (%).

The amino acid sequences share identities of 81.7% for SoH1-SoH2, 78.5% for SoH1-SoH3 and 77% for SoH2-SoH3. The topology of the maximum likelihood and the Bayesian trees are alike, therefore both results are displayed in one tree given both the support and bootstrap values, respectively. The phylogeny of cephalopod haemocyanin with the two isoforms of the gastropod *Haliotis tuberculata* as an outgroup is displayed in Figure 4.

**Figure 5:**
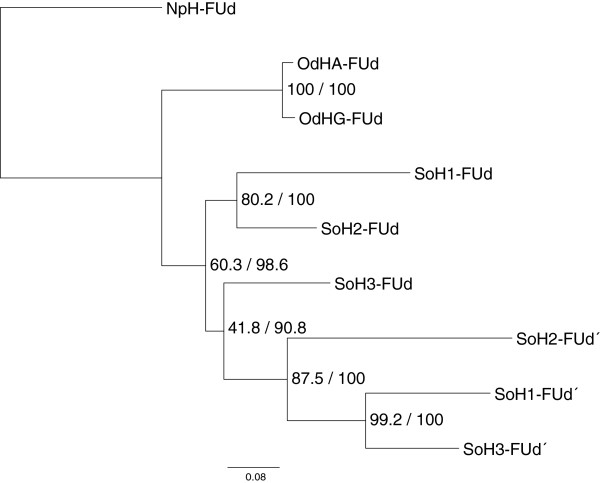
**Maximum likelihood tree of the amino acid sequences of FUd and FUd’.** The tree includes the FUd and FUd’ sequences of *Sepia officinalis* (SoH1-SoH3), *Enteroctopus dofleini* (OdHA, OdHG) and *Nautilus pompilius* (NpH) and was calculated with PhyML using the WAG + I + G model to illustrate the duplication of FUd/d’. The tree topology of the Bayesian tree calculated with MrBayes using the WAG + G + F model is similar. Therefore, support for the tree nodes is given by bootstrap values of 1,000 replicates and the consensus support (%).

Likewise, the phylogeny of FUd and FUd’ is shown in Figure 5.

The relaxed molecular clock with a mean evolutionary rate of 7.636 × 10^-4^ and a standard deviation of 0.237 revealed divergence times for the split of Tetrabranchiata-Dibranchiata of about 500 Mya and the split of Octobrachia-Decabrachia of about 267 Mya (Figure 2). Accordingly, the first duplication of cuttlefish haemocyanin occurred about 163 Mya and the second one about 100 Mya.

## Discussion

### Haemocyanin during embryogenesis

SoH3 is the most prominent haemocyanin isoform expressed in all analysed embryonic stages, whereas SoH1 and SoH2 expression starts during organogenesis (Lemaire 18–30). After hatching, the expression of SoH3 decreases drastically in the branchial gland (see also [22]). Hence, we could observe a shift of the haemocyanin isoforms during ontogeny. Accordingly, SoH3 represents an embryonic haemocyanin, whereas SoH1 and SoH2 are dominant in adult specimens. This is supported by the results of Decleir *et al.* (1970, 1971) [47,48], who detected electrophoretically distinct protein fractions of cuttlefish haemocyanin during ontogeny. However, Ruth *et al.* (1999) [63] could not detect haemocyanin in sections of the midgut gland and branchial gland of *Sepia* embryos younger than NAEF stage XVII (representing E2 of this study) by immunohistochemistry. In contrast, Wolf *et al.*[64] found evidence for haemocyanin in extracts of eggs and embryos (larger than 0.5 mm in mantle length) using immunoelectrophoresis. In vertebrates, a developmental shift of the respiratory protein is well studied [40,41] and also for invertebrates it has already been described [31,37,38]. Yet *Sepia officinalis* represents the only mollusc for which an embryonic form of haemocyanin has been published. Only a truncated haemocyanin form has so far been found in egg masses of *Biomphalaria glabrata*[65], a freshwater snail exhibiting haemoglobin as their respiratory protein. Additionally, we have some evidence for an embryonic haemocyanin isoform in the keyhole limpet *Megathura crenulata* according to protein analysis (Lieb *et al.*, unpublished).

SoH3 mRNA was also detected in yolk sac samples of *S. officinalis*, which at least in some way compares to humans, where the formation of blood cells also starts in the yolk sac [66]. Yet, this raises the question about the site of synthesis of haemocyanin during early embryogenesis. In *Cancer magister* the haemocyanin found in oocytes is of maternal origin and is subsequently replaced by an embryonic one [33]. In the gastropod *Haliotis asinina* no maternal haemocyanin mRNA could be detected, but the expression of haemocyanin started 9 h after fertilisation [67]. Considering that SoH3 mRNA is present in the embryo until hatching, it might rather represent an embryonic than a maternal haemocyanin, as maternal mRNA also requires a maternal translation machinery and is usually essential during early embryogenesis, but starts to be degraded partly from egg activation on and partly from the onset of zygotic transcription [68]. Decleir *et al.*[48] proposed the production of embryonic haemocyanin of cuttlefish in the perivitelline membrane of the yolk sac. In different cephalopods this site was indeed described as an enzymatically very active one yet only with respect to yolk digestion [69]. A production of embryonic haemocyanin in the yolk sac could very well also be the case as copper, the metal ion complexing oxygen in the haemocyanin molecule, is found in the yolk sac during early development and is only later on transferred into the embryo, whereas the total amount of copper remains constant during embryogenesis [70]. Furthermore, the yolk sac may be favoured for oxygen uptake in the early stages because of its greater surface compared to the embryo [71].

The expression site of haemocyanin is not uniform among the Mollusca and ranges from the rhogocytes/pore cells in the midgut gland in many gastropods [72-74] and *Nautilus* (a rather ancient cephalopod, which did not evolve branchial glands [63,75]), to the branchial glands in adult coleoid cephalopods. According to Beuerlein *et al.*[49] and Ruth *et al.*[75], the haemocyanin of cuttlefish embryos is also expressed in the midgut gland. This shift of the site of synthesis of haemocyanin during ontogeny may reflect an evolutionary shift from the midgut gland to the branchial gland in more highly evolved molluscs, namely recent coleoid cephalopods. The different expression patterns of the isoforms observed before hatching (E4) in the complete embryo (this study) and those observed only in gills (see [22]) may point to tissue differences in the expression and synthesis of the different isoforms at that developmental stage. Decleir *et al.*[48] proposed a shift of haemocyanin expression from the perivitelline membrane in early embryonic stages to the midgut gland when both tissues get in close contact and eventually to the branchial glands when they are fully developed. This was supported by Beuerlein *et al.*[49] at the mRNA level and immunologically by Ruth *et al.*[63], who concluded that these tissues undergo a switch of function in haemocyanin metabolism during ontogeny. However, as none of these studies distinguished between the different haemocyanin isoforms, the different isoforms cannot be assigned to specific expression sites for the time being.

The sequence data indicate that SoH3, which is the first isoform to be expressed during ontogeny, resulted from the first gene duplication and could be closest to the common ancestor of cuttlefish haemocyanin (Figure 4). The isoforms SoH1 and SoH2 resulted from a second gene duplication. SoH1, whose expression starts last, may represent the most derived isoform referring to the number of substitutions per site. As the differences in substitutions per site between the cuttlefish haemocyanin isoforms are fairly small, this assumption is speculative. On the other hand, molluscan haemocyanin generally has a nearly constant and only moderate evolutionary rate; therefore large differences between isoforms are not to be expected. In this line of thought, the dominating haemocyanin isoform as well as the site of synthesis of haemocyanin could represent a rather ancient state during embryogenesis. This insinuates that the ontogeny of cuttlefish haemocyanin, i.e. the shift of composition and expression site [49,63], may recapitulate its phylogeny, which is in accordance with Haeckel’s biogenetic law [76].

### Physiological aspects

Although the timing for the completion of the final site of haemocyanin synthesis, the branchial glands, would be well in line with the start of expression of SoH1 and SoH2, the change in expression site does not explain the need for a special embryonic version of the respiratory protein. SoH3 could additionally exhibit physiological properties, which might suit the embryonic situation better. Crustacean and mammalian embryonic respiratory proteins for example show different oxygen affinities compared to their adult equivalents to cope with the challenges during embryogenesis [41,43] and shifts of haemocyanin function and expression have also been discussed for gastropods [67,77]. Cuttlefish embryogenesis is characterised by hypoxic and hypercapnic conditions in the perivitelline fluid (PVF) towards the end of development [44] resulting from the egg capsule acting as a diffusion barrier [45,46]. According to Gutowska and Melzner [44], the *P*CO_2_ of the PVF in eggs of *Sepia officinalis* increases during embryogenesis from 0.13 kPa to 0.41 kPa (0.04 kPa in ambient seawater), corresponding to a decrease in pH from 7.72 to 7.23.

The O_2_ affinity of cephalopod haemocyanin is strongly pH-dependent [29] and could therefore be influenced by increasing carbon dioxide pressure (*P*CO_2_) towards the end of embryogenesis, which is estimated to rise to 0.6–0.8 kPa at the end of embryogenesis [44]. Furthermore, respiratory proteins of organisms living under hypoxia, i.e. the oxygen minimum zones (OMZ), express higher oxygen affinities in order to cope with the reduced oxygen availability [78,79]. The three haemocyanin isoforms of *S. officinalis* exhibit different theoretical isoelectric points (SoH1: 5.79, SoH2: 5.85, SoH3: 5.94) and different amounts of histidine residues (SoH1: 168, SoH2: 179, SoH3: 173) pointing at different buffer capacities and O_2_ affinities. These could come in handy during embryogenesis as abiotic conditions change, however clearly require further analysis before conclusions on the function of Hc3 can be reached.

### Phylogenetic aspects

The embryonic haemocyanin of cuttlefish already represents the third isoform found in *S. officinalis.* It has the same genetic structure (presence of signal peptide, number of FUs, exon/intron structure) as found in the other isoforms. As mentioned above, it could also be the isoform most similar to the common ancestor of cuttlefish haemocyanin referring to the number of substitutions per site (Figure 4). The ambiguities that we could not clarify with the help of the genomic data may indicate SNPs between alleles. The unresolved positions observed in the cDNA sequence only could therefore be a result of pooled samples or even propose the existence of more than three isoforms, as fragments differing from all haemocyanin sequences found so far in the cuttlefish were also found at the genomic level.

Referring to the number of oxygen binding sites and the molecular weight of haemocyanin proteins, it has been suggested that *Nautilus* and *Octopus* exhibit 7 FUs whereas the haemocyanin of *Sepia* and *Loligo* consists of 8 FUs [1]. As molluscan haemocyanin evolved from subsequent gene duplications, an additional duplication could have occurred within the Dibranchiata. This is supported by molecular data for *Spirula spirula* and *Sepia officinalis* SoH1, SoH2 and now also SoH3, which indicate a duplication of FUd/d’ [11,13] (DeGeest, unpublished, see also Figure 5) that is present in neither *Nautilus* nor *Octopus*[6,9]. It is rather unlikely that the duplication occurred independently in all four haemocyanins. Furthermore, the tree topology shown in Figure 5 supports the assumption that the FU duplication was prior to the isoform duplication in cuttlefish, as orthologues of both FUs (e.g. SoH1_FUd’, SoH2_FUd’, SoH3_FUd’) and not paralogues (e.g. SoH1_FUd, SoH1_FUd’) are assigned to each other. Referring to FUd, SoH1 is more closely related to SoH2, whereas FUd’ indicates that SoH1 is more closely related to SoH3. These differences in topology between the two groups of orthologues resulting from different substitution rates also point to a duplication into FUd/d’ much earlier than the duplication of the isoforms. This would support the assumption of FUd’ being characteristic for Dibranchiata [13]. However, no sequence information for other groups within the Dibranchiata, e.g. squids, is available so far.

In contrast to the duplication into FUd/d’ the duplication of the complete haemocyanin molecule occurred independently in several molluscs (*Haliotis tuberculata*[16,17], *Megathura crenulata*[19]*, Nucula nucleus*[8], *Enteroctopus dofleini*[13], *Sepia officinalis* (DeGeest, unpublished)). Compared to the rather recent haemocyanin duplication in *Enteroctopus* about 13 Mya, the first duplication of the cuttlefish haemocyanin occurred about 163 Mya resulting in the ancestor of SoH3 (Figure 2). The second duplication about 100 Mya led to the progenitor of SoH1 and SoH2. These calculated divergence times correspond well to fossil data and previous results [6,12,13].

## Conclusion

The haemocyanin isoform 3 in cuttlefish represents an embryonic version of the respiratory pigment as it is expressed on a high level during embryogenesis, but is downregulated after hatching. Concerning its physiological properties only speculations based on the pI deduced from the primary structure can be done so far. The structural organisation of SoH3 with respect to the exon-intron structure and the duplication of FUd/FUd’ reflects the situation found in the other cuttlefish haemocyanin isoforms. Compared to these, SoH3 probably represents a rather ancient state with respect to its sequence and expression site, thereby leading to the assumption that phylogeny might be recapitulated during ontogeny.

## Abbreviations

SoH1: *Sepia officinalis* haemocyanin isoform 1; SoH2: *Sepia officinalis* haemocyanin isoform 2; SoH3: *Sepia officinalis* haemocyanin isoform 3; OdHA: *Enteroctopus dofleini* haemocyanin type A; OdHG: *Enteroctopus dofleini* haemocyanin type G; HtH1: *Haliotis tuberculata* haemocyanin isoform 1; HtH2: *Haliotis tuberculata* haemocyanin isoform 2; NpH: *Nautilus pompilius* haemocyanin; FU: Functional unit; SU: Subunit; Bp: Basepairs; Aa: Aminoacids; Mya: Million years ago; PO2: Oxygen partial pressure; PCO2: Carbondioxide partial pressure; UTR: Untranslated region; ORF: Open reading frame; COX: Cytochrome c oxidase; E1: Embryonic stage 1 (Lemaire 21–24); E2: Embryonic stage 2 (Lemaire 25–26); E3: Embryonic stage 3 (Lemaire 26–27); E4: Embryonic stage 4 (Lemaire 28–29); H: hatchling (Lemaire 30, hatchlings); A1: Adult stage.

## Competing interests

The authors declare that they have no competing interests.

## Authors’ contributions

AT and FCM conceived and designed the study. AT, MO and BL participated in the sequencing, sequence analysis and/or interpretation. AT and FCM carried out the expression experiments and/or data analysis. AT drafted the manuscript, and FCM and BL revised it critically. All authors read and approved the final manuscript.
